# Characterization of Mass Shootings by State, 2014-2022

**DOI:** 10.1001/jamanetworkopen.2023.25868

**Published:** 2023-07-26

**Authors:** Leslie M. Barnard, Erin Wright-Kelly, Ashley Brooks-Russell, Marian E. Betz

**Affiliations:** 1 Department of Epidemiology, Colorado School of Public Health, University of Colorado Anschutz Medical Campus, Aurora; 2 Injury and Violence Prevention Center, Colorado School of Public Health, University of Colorado Anschutz Medical Campus, Aurora; 3 Department of Emergency Medicine, University of Colorado School of Medicine, University of Colorado Anschutz Medical Campus, Aurora

## Abstract

This case series investigates the rates of mass shootings, along with injuries and deaths, by US state and shooting type.

## Introduction

The US has more than 10 times the number of mass shooting events as other developed countries.^1^ Mass shootings in the US have increased in frequency, with more than half occurring since the year 2000.^2^ These events have a direct toll on individuals injured or killed, as well as a psychological impact on families, friends, and society.^3^

Little research has examined the types and distribution of mass shooting events across the US.^4^ A geographic analysis by type may inform if specific events have disproportionately occurred in particular states or regions of the US. This may generate hypotheses about the contextual (policy, environmental, or sociocultural) factors that may be associated with the distribution of types of mass shooting events and may suggest recommendations for tailored prevention. The purpose of this study was to examine state rates of mass shooting event types and total injuries and deaths in the US.

## Methods

The Gun Violence Archive, a database mostly used for research,^5^ defines a mass shooting as an incident with 4 or more individuals shot or killed, not including the shooter. This case series used data from a 9-year period (January 1, 2014, to December 31, 2022) to calculate cumulative incidence rates of mass shooting event types based on incident characteristics (Figure) and the total number of individuals injured and killed per 1 000 000 people. We also calculated state-level counts and rates for the most common event types. Rates were calculated using population estimates from the US census from 2014 to 2022 and displayed on state-level heat maps.

**Figure.**
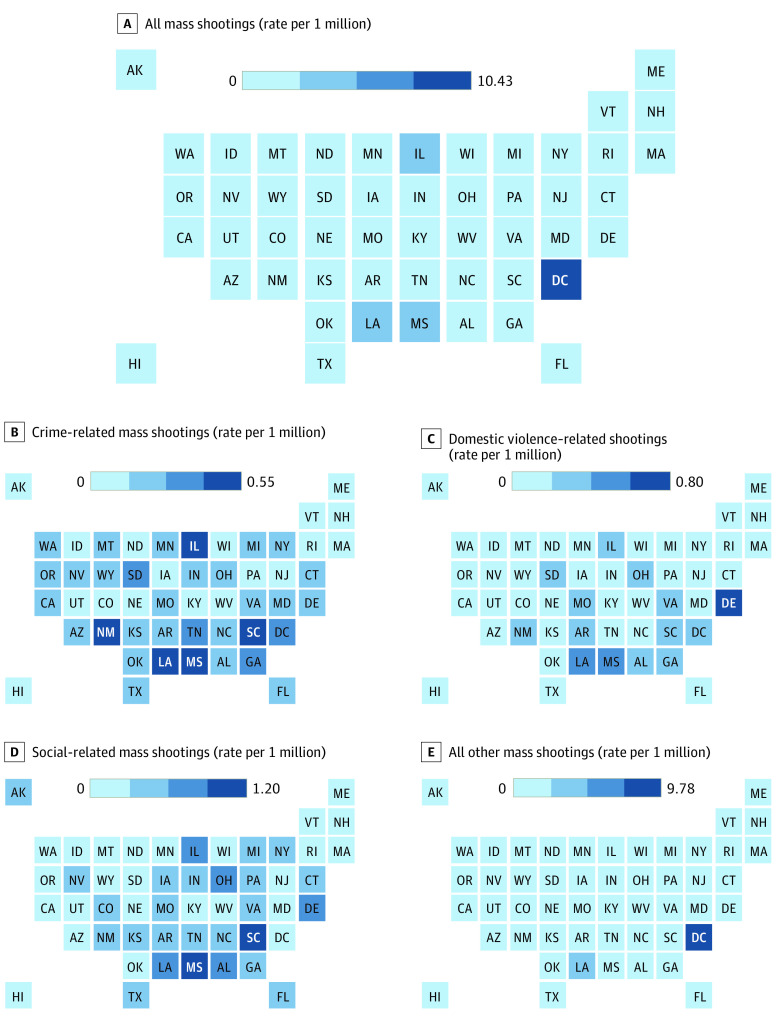
Cumulative Incidence of Mass Shootings, 2014-2022 Heat maps are presented of the cumulative incidence rate of mass shootings and injuries and deaths from mass shootings by state, 2014 to 2022. Subcategories were gang or drug involvement, armed robbery, carjacking, murder or suicide, and home invasion for crime-related events; domestic violence, family annihilation, kidnapping, or involving a child for domestic violence–related events; bar or club and house party for social-related events; and terrorism, spree shooting, hate crimes, and others for other events.

## Results

From 2014 to 2022, there were 4011 mass shootings, ranging from zero events in Hawaii and North Dakota to 414 events in Illinois. For these 9 years, one-third (27.3%) were social-related mass shootings, 15.8% were crime related, 11.1% were domestic violence (DV) related, 1.4% were school or work related, and 52.0% were not a part of these categories (Table). There was a median of 45 mass shootings per state for all states and the District of Columbia (mean, 78.6). A total of 21 006 people were killed or injured (Table).

**Table.** Incidence of Mass Shootings, Deaths, and Injuries, 2014-2022

State	Population, No.^a^	Mass shooting type										Total injuries and deaths	
		Total		Crime related		DV related		Social related		Other			
		No.	Rate^b^	No.	Rate^b^	No.	Rate^b^	No.	Rate^b^	No.	Rate^b^	No.	Rate^b^
AL	44 378 527	103	2.32	11	0.25	13	0.29	31	0.70	51	1.15	505	11.38
AK	6 622 309	5	0.76	0	0.00	1	0.15	2	0.30	2	0.30	24	3.62
AZ	63 785 927	45	0.71	9	0.14	6	0.09	19	0.30	14	0.22	249	3.90
AR	27 052 194	44	1.63	7	0.26	7	0.26	10	0.37	23	0.85	257	9.50
CA	352 688 428	367	1.04	79	0.22	36	0.10	98	0.28	193	0.55	1874	5.31
CO	50 837 935	60	1.18	5	0.10	6	0.12	21	0.41	28	0.55	327	6.43
CT	32 316 813	28	0.87	7	0.22	0	0.00	12	0.37	10	0.31	138	4.27
DE	8 734 043	19	2.18	2	0.23	7	0.80	7	0.80	5	0.57	85	9.73
DC	6 137 207	64	10.43	2	0.33	2	0.33	1	0.16	60	9.78	321	52.30
FL	190 016 591	237	1.25	35	0.18	30	0.16	59	0.31	129	0.68	1345	7.08
GA	94 517 232	155	1.64	32	0.34	20	0.21	45	0.48	68	0.72	769	8.14
HI	12 863 400	0	0	0	0	0	0	0	0	0	0	0	0
ID	15 912 416	2	0.13	2	0.13	0	0	0	0	0	0	11	0.69
IL	114 792 734	414	3.61	59	0.51	27	0.24	80	0.70	260	2.26	2073	18.06
IN	60 357 532	85	1.41	16	0.27	8	0.13	33	0.55	34	0.56	422	6.99
IA	28 395 696	18	0.63	2	0.07	2	0.07	12	0.42	5	0.18	92	3.24
KS	26 266 712	22	0.84	5	0.19	0	0	13	0.49	6	0.23	122	4.64
KY	40 185 945	48	1.19	4	0.10	5	0.12	11	0.27	28	0.70	244	6.07
LA	41 834 823	179	4.28	23	0.55	18	0.43	37	0.88	108	2.58	917	21.92
ME	12 134 108	3	0.25	1	0.08	2	0.16	0	0	1	0.08	14	1.15
MD	54 563 993	120	2.20	14	0.26	8	0.15	12	0.22	91	1.67	563	10.32
MA	61 982 752	34	0.55	6	0.10	3	0.05	12	0.19	16	0.26	162	2.61
MI	89 897 371	129	1.43	14	0.16	13	0.14	38	0.42	70	0.78	618	6.87
MN	50 406 473	49	0.97	11	0.22	4	0.08	10	0.20	29	0.58	258	5.12
MS	26 760 340	78	2.91	12	0.45	12	0.45	32	1.20	26	0.97	398	14.87
MO	55 082 568	126	2.29	12	0.22	17	0.31	25	0.45	75	1.36	609	11.06
MT	9 591 297	4	0.42	2	0.21	0	0	1	0.10	1	0.10	19	1.98
NE	17 346 302	15	0.86	2	0.12	1	0.06	4	0.23	8	0.46	79	4.55
NV	27 119 354	31	1.14	7	0.26	4	0.15	10	0.37	13	0.48	657	24.23
NH	12 235 284	1	0.08	0	0	0	0	0	0	1	0.08	4	0.33
NJ	81 058 390	93	1.15	9	0.11	5	0.06	15	0.19	67	0.83	459	5.66
NM	18 900 254	24	1.27	8	0.42	5	0.26	9	0.48	7	0.37	115	6.08
NY	177 155 566	186	1.05	26	0.15	12	0.07	61	0.34	101	0.57	910	5.14
NC	92 971 556	115	1.24	17	0.18	16	0.17	38	0.41	52	0.56	561	6.03
ND	6 857 698	0	0	0	0	0	0	0	0	0	0	0	0
OH	105 197 605	153	1.45	21	0.20	22	0.21	66	0.63	59	0.56	807	7.67
OK	35 518 477	29	0.82	7	0.20	6	0.17	7	0.20	12	0.34	139	3.91
OR	37 353 493	22	0.59	6	0.16	1	0.03	7	0.19	10	0.27	121	3.24
PA	115 724 473	191	1.65	12	0.10	20	0.17	37	0.32	130	1.12	927	8.01
RI	9 629 156	4	0.42	1	0.10	0	0	1	0.10	2	0.21	22	2.28
SC	45 535 409	103	2.26	20	0.44	11	0.24	46	1.01	37	0.81	550	12.08
SD	7 896 125	4	0.51	3	0.38	2	0.25	0	0	0	0	20	2.53
TN	61 033 316	124	2.03	21	0.34	12	0.20	31	0.51	67	1.10	621	10.17
TX	257 089 529	270	1.05	60	0.23	44	0.17	87	0.34	103	0.40	1560	6.07
UT	28 424 830	6	0.21	3	0.11	2	0.07	1	0.04	1	0.04	30	1.06
VT	5 683 707	1	0.18	0	0	1	0.18	0	0	0	0	4	0.70
VA	76 560 754	97	1.27	15	0.20	19	0.25	27	0.35	43	0.56	493	6.44
WA	67 325 769	45	0.67	17	0.25	6	0.09	14	0.21	13	0.19	215	3.19
WV	16 288 106	5	0.31	1	0.06	2	0.12	2	0.12	1	0.06	25	1.53
WI	52 374 030	53	1.01	7	0.13	9	0.17	12	0.23	27	0.52	267	5.10
WY	5 226 119	1	0.19	1	0.19	0	0	0	0	0	0	4	0.77
Total	2 938 620 668	4011	1.36	636	0.22	447	0.15	1096	0.37	2087	0.71	21 006	7.15
Mean (SD)	57 620 013	79 (91.4)	NA	12 (15.9)	NA	9 (9.8)	NA	21 (24.1)	NA	41 (52.5)	NA	412 (473.5)	NA
Median (IQR)	40 185 945	45 (6-124)	NA	7 (2-17)	NA	6 (1-13)	NA	12 (2-37)	NA	23 (2-67)	NA	257 (30-618)	NA

Abbreviation: NA, not applicable.

^a^ Columns may not add up to total owing to overlapping categories. Populations are based on US Census estimates from 2014 to 2022.

^b^ All rates are per 1 000 000 individuals.

The rate of mass shootings per 1 000 000 people was highest in the District of Columbia (10.4 shootings), followed by much lower rates in Louisiana (4.2 mass shootings) and Illinois (3.6 mass shootings), the states with the next 2 highest rates (Table). Geographical analysis of mass shooting events showed clustering around the southeast region of the US and Illinois (Figure). Crime-, social-, and DV-related mass shootings followed a similar pattern, while mass shootings that were not part of these categories were more evenly distributed across the US.

## Discussion

This case series examined the number and rate of mass shooting events (including injuries and deaths) by type of event and US state differences. While results demonstrated state-level differences in rates of mass shootings, findings were limited given use of 1 database with a broad definition of mass shootings. Definitions that include only deaths or events not associated with crime or only indiscriminate mass public shootings may produce different results.^6^ Future research should assess socioeconomic, political, cultural, and demographic factors that may be associated with incidents of mass shootings across states and address how state policies, contextual factors, and social determinants of health may be associated with mass shooting incident types.

This study of mass shootings examined the burden of and geographic differences between types of mass shootings in the US. The most common specific event type was crime-related mass shootings. Crime-, social-, and DV-related mass shootings followed a similar pattern, with clustering around the southwest. These findings should be used to inform research and state-level prevention strategies.

## Notes

Supplement.
Data Sharing Statement
