# Supplementary material for: Characterization of Mass Shootings by State, 2014-2022
Source: JAMA Netw Open. 2023 Jul 26;6(7):e2325868. doi: 10.1001/jamanetworkopen.2023.25868 (PMC10372703; doi:10.1001/jamanetworkopen.2023.25868)
Supplement: Supplement. — Data Sharing Statement [file jamanetwopen-e2325868-s001.pdf]

Data Sharing Statement

Barnard. Characterization of Mass Shootings by State, 2014-2022. *JAMA Netw Open*. Published online July 26, 2023. doi:10.1001/jamanetworkopen.2023.25868

Data
Data available: Yes
Data types: Other (please specify)
Additional Information: All data is publically available.
How to access data: https://www.gunviolencearchive.org/
When available: beginning date: 01-01-2014, end date: 12-31-2022

Supporting Documents
Document types: None

Additional Information
Who can access the data: Anyone can access this data.
Types of analyses: Analytic code is available upon request from the corresponding author.
Mechanisms of data availability: with investigator support
